# Evaluation of the Second Follicular Wave Phenomenon in Natural Cycle Assisted Reproduction: A Key Option for Poor Responders through Luteal Phase Oocyte Retrieval

**DOI:** 10.3390/medicina55030068

**Published:** 2019-03-14

**Authors:** Konstantinos Sfakianoudis, Mara Simopoulou, Evangelos Maziotis, Polina Giannelou, Petroula Tsioulou, Anna Rapani, Agni Pantou, Konstantina Petroutsou, Irene Angeli, Efthymios Deligeoroglou, Michael Koutsilieris, Konstantinos Pantos

**Affiliations:** 1 Centre for Human Reproduction, Genesis Athens Clinic, 15232 Chalandri, Greece; sfakianosc@yahoo.gr (K.S.); lina.giannelou@gmail.com (P.G.); agnipantos@gmail.com (A.P.); dinapetroutsou@hotmail.com (K.P.); renaangelis@yahoo.co.uk (I.A.); info@pantos.gr (K.P.); 2 Department of Physiology, Medical School, National and Kapodistrian University of Athens, 11527 Athens, Greece; vagmaziotis@gmail.com (E.M.); petroulatsi@yahoo.gr (P.T.); rapanianna@gmail.com (A.R.); mkoutsil@med.uoa.gr (M.K.); 3 Assisted Conception Unit, 2nd Department of Obstetrics and Gynaecology, Aretaieion Hospital, Medical School, National and Kapodistrian University of Athens, 11526 Athens, Greece; edeligeo@aretaieio.uoa.gr; 4 Department of Physiology, Medical School, Democritus University of Thrace, 68100 Alexandroupolis, Greece

**Keywords:** second follicular wave, LuPOR, poor responders, natural cycle

## Abstract

*Background*: Emergence of Luteal Phase Oocyte Retrieval (LuPOR) may revolutionize the practice regarding the time-sensitive nature of poor responders ascertaining a higher number of oocytes, in a shorter amount of time. This may be especially important in view of employing the approach of natural cycles for Poor Responders. We suggest the acronym LuPOR describing the clinical practice of luteal phase oocyte retrieval. The aim of the study is to offer insight regarding the identity of LuPOR, and highlight how this practice may improve management of the special subgroup of poor responders. *Materials and Methods*: The present retrospective observational clinical study includes the collection and statistical analysis of data from 136 poor responders who underwent follicular oocyte retrieval (FoPOR) and subsequent LuPOR in natural cycles, during their In Vitro Fertilization (IVF) treatment, from the time period of 2015 to 2018. All 136 participants were diagnosed with poor ovarian reserve (POR) according to Bologna criteria. The 272 cycles were categorized as follows: 136 natural cycles with only FoPORs (Control Group) and 136 natural cycles including both FoPORs and LuPORs. *Results*: Our primary results indicate no statistically significant differences with regards to the mean number of oocytes, the maturation status, and fertilization rate between FoPOR and LuPOR in natural cycles. Secondarily, we demonstrate a statistically significant higher yield of oocytes (2.50 ± 0.78 vs. 1.25 ± 0.53), better oocyte maturity status (1.93 ± 0.69 vs. 0.95 ± 0.59) and higher fertilization rate (1.31 ± 0.87 vs. 0.61 ± 0.60) in natural cycles including both FoPOR and LuPOR, when compared to cycles including only FoPOR. *Conclusion*: Our study may contribute towards the establishment of an efficient poor responders’ management through the natural cycle approach, paving a novel clinical practice and ascertaining the opportunity to employ oocytes and embryos originating from a luteal phase follicular wave.

## 1. Introduction

Ovarian function was firstly observed through histologic and endocrinologic methods [1]. It was almost four decades later that follicles were visualized and their development was observed. The prevailing theory at that time was a single follicular wave and the development of a single leading follicle during each cycle [2]. A decade later more than two dominant follicles were detected in each cycle altering the scientific approach of the field [3], a phenomenon concurrently observed in animal models such as cattle [4] or bovine [5]. A thorough literature search from the same group cemented the phenomenon of subsequent follicular waves during a cycle [6]. This newly founded knowledge was soon introduced in assisted reproduction techniques (ART). In Vitro fertilization (IVF) experts encompassed it within their protocols leading to an alternative approach of luteal phase oocyte retrieval (LuPOR) towards assisting infertile women undergoing IVF [7] with a special focus to those presenting with poor ovarian response (POR).

In 1983, Garcia et al. reported for the first time in bibliography the different ovarian response and estradiol (E_2_) levels to ovarian stimulation protocols and categorized the patients in groups highlighting the subgroup of patients with POR and the need for a different treatment approach [8]. Since then, a great number of scientific groups, representing different perspectives in the ART world, investigated the group of patients with POR regarding the pathophysiology of this phenomenon, its clinical characterization and possible treatments [9]. It is notable that, between 2000 and 2010, six reviews on this topic similarly concluded that there is a lack of well-defined criteria to successfully identify these patients, and a clear need for an internationally accepted definition [10,11,12,13,14]. Addressing these issues along with introducing globally adopted guidelines remains the goal in improving treatment outcomes for this challenging group of ART patients [9]. In clinical practice, there are various methods in order to assess ovarian reserve such as evaluation of Follicle-Stimulating Hormone (FSH) and E_2_ levels on day 3 of the menstrual cycle, anti-Müllerian hormone (AMH) levels and antral follicle count (AFC) [15]. Age, however, remains the most important factor as in recent years, and there has been a tendency to delay pregnancy for social and/or economic reasons, resulting in an increasing number of women of an advanced age seeking infertility treatment [16]. This heterogeneity in assessment is still cause for discrepancies in categorization and optimal treatment. In 2011, a consensus was reached in order to clearly report the minimal criteria required to define POR. These are known as the Bologna criteria [9]. The Bologna criteria are the most commonly employed criteria to identify these patients. However, practices fail to rely on evidence-based treatment indicating the lack of an international consensus on this topic [17]. These challenges prompted a group of clinicians in late 2017 to proceed with suggesting a new model and a tool for handling these low prognosis patients. The POSEIDON (Patient-Oriented Strategies Encompassing Individualized Oocyte Number) group employs the number of oocytes required to obtain one euploid embryo for transfer in each patient as a pragmatic endpoint for categorizing the IVF patients in subgroups [18].

The major challenge of every ART specialist and IVF clinic worldwide is to apply the optimal treatment protocol to these heterogeneously diagnosed patients with low prognosis, in order to achieve a positive result and deliver a healthy baby to the infertile couple. A broad range of protocols have been proposed, aiming to increase respective ovarian response, such as Gonadotropin-Releasing Hormone (GnRH) analogue protocols [14,19], combination of gonadotropins [20,21,22,23], protocols with clomiphene citrate and gonadotropins [23] and protocols that employ human Chorionic Gonadotropin (hCG), melatonin, myo-inositol, baby aspirin, low molecular weight heparin and Dehydroepiandrosterone (DHEA) [24,25,26,27,28,29,30,31,32]. The antagonist protocol, the microdose flare protocol and the long downregulation protocol have been identified as the most popular interventions employed [33].

A different auspicious approach has been described through the employment of natural cycles for poor responders [13]. Let us not forget that the first successful IVF was performed on a natural cycle. On a more recent note, there is bibliographic evidence indicating a small but clear and consistent improvement of the results when the approach of natural cycles is recruited [34,35] in extreme POR patients. However, large prospective randomized controlled studies are required. Based on this, two different schools of thought have been formed regarding the optimal approach for poor responders. Some IVF experts believe that stimulation protocols ascertain the optimal results for POR treatment while others opt for the natural cycle approach, since they anticipate an equally poor ovarian response with any attempt of pharmaceutical stimulation. Regarding the natural cycle approach, it can be further distinguished in two subgroups. On one hand, performance of a natural cycle is concluded followed by an impending single embryo transfer, or, on the other hand, repeated natural cycles and subsequent cryopreservation of the resulting embryos describes the “freeze and collect” or “single embryo banking approach”. In this case, the patient gradually builds a cohort of cryopreserved embryos in storage. At the appropriate time, a cycle—including these cryopreserved embryos—is planned leading to an embryo transfer employing the best embryos [36,37]. The most important parameter for POR patients of advanced maternal age is time [38]. Acknowledging this fact, and exploring ways to ascertain time and oocyte yield, efficient treatment for POR has focused on the phenomenon of the Second Follicular Wave (SFW) and directed our efforts into the best way of integrating this practice into the strategy decided and performed for these patients.

In an effort to unlock the full potential of the SFW by enabling the highest possible oocyte yield from both phases, modified double controlled ovarian stimulation (COS) protocols have been recruited [7]. The term “Duo Stim” has been suggested in describing this approach successfully [39,40]. Various studies attempted to compare the IVF results of double ovarian stimulation within the same menstrual cycle. The majority of them indicate that following DuoStim there are no statistically significant differences in the mean number of cumulus–oocyte complexes for IVF and mature MII oocytes retrieved between follicular phase and luteal phase, [7,39,40,41,42]. Further to that, it seems that oocytes retrieved from luteal phase stimulation present with the same maturation and fertilization rate with those of follicular phase stimulation [43,44,45]. This evidence concludes that high quality oocytes may be retrieved following the double stimulation protocol [45,46]. In the same line, follow recently reported results regarding the consensus on LuPOR from the embryology laboratory perspective. These include the number yielded and the fertilization rate of the oocytes [45], the ability of the respective zygotes to develop into cleavage stage embryos [47] along with the subsequent blastocyst stage development rate [46], as well as the chromosomal status of the embryos originating from SFW oocytes [39]. These studies report no statistically significant differences between stimulated cycles both in the follicular and luteal phase.

In contrast to the majority of the studies with regards to stimulated cycles, little is known about the performance, the dynamic and quality or even the existence of luteal phase oocytes and embryos in comparison to those originating from the follicular phase during natural cycles in extreme POR patients. It is evident from current literature that the lack of competent criteria and a consensus on the optimal treatment for poor responders, urge the scientific community of ART to explore options aiming to ascertain improved management. This fact renders the present study timely and essential and constitutes the driver of this study. The aim of this article is to open a new line of research dedicated in thoroughly understanding the true place of the SFW in IVF through LuPOR, while, concurrently, fuel investigation towards defining optimal management for the special subgroup of poor responders.

## 2. Materials and Methods

### 2.1. Study Population

**Figure 1:**
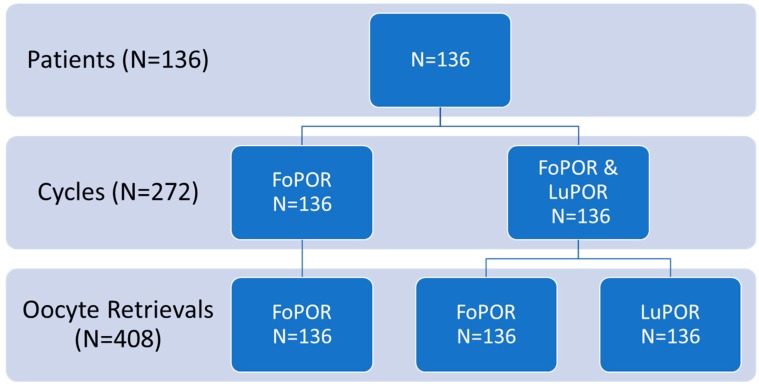
Classification of oocyte retrievals according to each phase and cycle performed.

A total of 153 women met the inclusion criteria and signed the informed consent for participating in this study. Seventeen out of 153 (11.11%) cancelled due to lack of adequate follicle development, natural ovulation or failure to attain to the appointment. The remaining 136 patients underwent 408 Oocyte Retrievals (OR) corresponding to 3 oocyte retrievals per patient. Each patient underwent two cycles, one cycle corresponding to natural follicular phase oocyte retrieval (FoPOR) only serving as the control group, and the other corresponding to both natural follicular and natural luteal phase oocyte retrievals (FoPOR and LuPOR). Consequently, the control group of this study corresponds to the same patient group. It should be noted that the two cycles the 136 patients underwent refer to consecutive menstrual cycles; the first one including FoPOR alone and the second one including both FoPOR and LuPOR. A graphic representation of the number of cycles and oocyte retrievals is presented in Figure 1.

All participants were diagnosed with POR according to Bologna criteria as they fulfilled at least 2 out of 3 requirements: Maternal age ≥40, previous POR in a stimulation cycle (less than 3 oocytes received) and/or AMH levels <1.1 ng/mL. In fact, most of the patients had presented with one or zero oocytes retrieved in previous stimulation cycles. Male factor infertility was excluded in this study population, as the sole cause of infertility for this group of patients was advanced maternal age and reduced ovarian reserve.

### 2.2. Natural Cycle Protocol, Oocyte Retrieval and Fertilization

All patients of the study group identified as poor responders according to Bologna criteria. Following the first appointment, baseline levels of FSH, luteinizing hormone (LH) and E_2_ were recorded and patients with FSH levels over 15 mIU/mL were recommended to undergo a series of natural cycles. Follicular growth was monitored via transvaginal ultrasonography on the eighth day of the cycle along with daily recordings of serum levels of LH and E_2_. When the leading follicle met the maturity criteria: diameter of >15 mm and serum E_2_ levels of >100 pg/mL, at that time an intramuscular injection of 5000 IU of hCG or recombinant hCG was administered subcutaneously for ovulation triggering. Follicular aspiration was performed 36 h following hCG administration employing an ultrasonically guided vaginal probe with or without the need for sedation or anesthesia.

Oocytes were cultured under standard laboratory conditions and 40 h post hCG, insemination by Intracytoplasmic Sperm Injection (ICSI) was employed. Sixteen to eighteen hours post insemination fertilization assessment was performed and zygotes were evaluated. Normally, fertilized zygotes identifying 2 pronuclei were cryopreserved until collection of an optimal number of embryos was achieved. Previous clinical observations within our practice highlighted this category of patients presenting with new follicle recruitment during the luteal phase as serum levels of E_2_ remained high in the luteal phase and at the onset of the subsequent follicular phase. These patients were advised to undergo a second monitoring for follicular growth employing only transvaginal ultrasonography seven days post oocyte retrieval. When a new leading follicle of >18 mm was observed, one intramuscular injection of 5000 IU of hCG or subcutaneous injection of 250 μg of hCG was administered again for ovulation triggering and 36 h later LuPOR was performed. Identical laboratory protocols as described above led to a second round of cryopreserved embryos within the same cycle.

## 3. Statistical Analysis

Statistical analysis was performed employing R statistical programming language via the RStudio interpreter (Boston, MA, USA). Normality of the distribution was evaluated via the Shapiro–Wilks test. Due to the fact that the distributions of most parameters were not normal—with the exception of the parameters of the number of oocytes retrieved, MII oocytes and number of 2PN zygotes—the Wilcoxon rank-sum test (Mann–Whitney U) instead of the Student’s *t*-test was preferred to examine potential differences between groups in the aforementioned cases. If the distributions of both groups examined were normal, the Student’s *t*-test was employed.

## 4. Results

### 4.1. Patients’ Characteristics

The hormonal profile of the patients included data on levels of FSH, AMH, E_2_, and progesterone, reflecting the cycle’s identity. These data along with information on the patients’ age is presented in Table 1. The parameters examined herein aim to depict the performance of each cycle. For this purpose, data from the IVF laboratory were sourced including the number and the oocytes’ maturity status, along with their fertilization dynamic following ICSI. With regards to the maturity level of the oocytes obtained, the oocytes were classified as mature oocytes (MII) having extruded the first polar body, immature oocytes with no polar body extrusion (MI), and immature oocytes with a germinal vesicle present (GV). The number of abnormal oocytes retrieved are separately accounted for. With regards to the fertilization status, the study presents data on number of zygotes describing the normally fertilized oocytes bearing two pronuclei (2PN) (zygotes), the number of unfertilized oocytes (0PN), the number of abnormally fertilized oocytes (3PN), and finally the number of lysed oocytes following ICSI. Further to the above, in this study, an 11.11% cancellation rate of oocyte retrievals was noted as some patients presented with inadequate follicle development, ovulated naturally, or failed to show to appointment.

### 4.2. Comparison of FoPORs and LuPORs during the Same Unstimulated Natural Menstrual Cycle

The first scale of comparison involved the FoPORs data versus the subsequent data LuPORs data. This comparison aimed to identify differences and similarities between the oocytes retrieved from the two phases in the same patient and during the same unstimulated natural menstrual cycle. The parameters studied as mentioned above included a comparison between the number of oocytes yielded, their maturity status, and the respective fertilization rate. These results are presented in Table 2. Follicular and luteal phase oocyte retrieval during the same menstrual cycle presented with no statistically significant difference regarding any of the parameters studied (Table 2).

**Figure 2:**
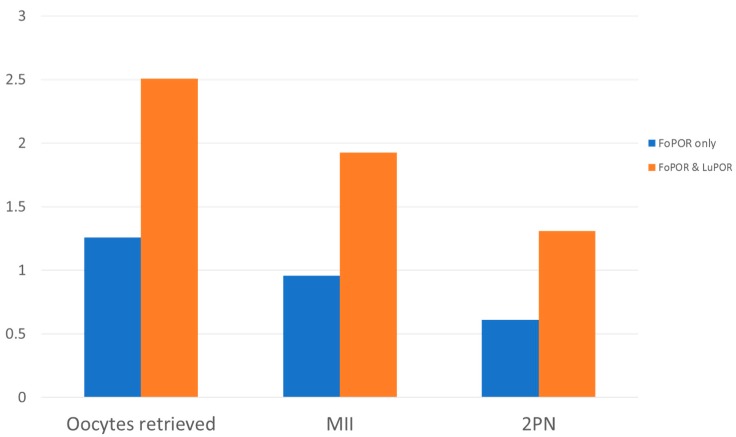
Number of oocytes retrieved, maturity status and two pronuclei (2PN) zygotes in control group (Follicular Phase Oocyte Retrieval-FoPOR only) and in the study group (FoPOR and Luteal Phase Oocyte Retrieval-LuPOR).

Secondarily, we compared the FoPOR data in cycles that did not follow with LuPOR (FoPORs only), to the accumulative data of cycles including both FoPORs and subsequent LuPORs. All comparisons were in the context of natural unstimulated cycles. These results are presented in Table 3. Natural cycles including both FoPORs and LuPORs presented with a statistically significant higher average number on four of the parameters studied, namely: number of oocytes retrieved (2.50 ± 0.78 vs. 1.25 ± 0.53), MII oocytes (1.93 ± 0.66 vs. 0.95 ± 0.59) 2PN zygotes (1.31 ± 0.87 vs. 0.61 ± 0.60) and lysed oocytes (0.14 ± 0.35 vs. 0.07 ± 0.26) (Table 3). A graphic representation of the number of oocytes retrieved, number of MII oocytes and 2PN embryos are presented in Figure 2.

## 5. Discussion

The reveal of the SFW and the emergence of LuPOR lead to a new trend revealing a new strategy for the special group of poor responders [3]. Delineation of the continuous follicular wave and its implementation in IVF through LuPOR have successfully created alternatives for Advanced Maternal Age (AMA) and Diminished Ovarian Reserve (DOR) patients [40]. However, all studies reporting on SFW within the IVF scope refer to hormonal stimulation approaches. This approach, known as “Double Ovarian Stimulation” or “Duo Stim” [40,48], seems to relieve the time related patients’ stress providing a higher number of oocytes retrieved in a shorter period, when compared to the conventional stimulation protocol in follicular phase [49].

As far as the embryo is concerned, it seems that MII oocytes retrieved following stimulation in either follicular or luteal phase may result in the same number of cleaved embryos [45] of similar developmental potential, successfully reaching the blastocyst stage on days 5, 6 and 7 [39]. Oocytes originating from both phases could lead to a similar number of top-quality embryos [39,44]. Numerous studies examined the blastocysts biopsied for Preimplantation Genetic Screening (PGS) following DuoStim, resulting in the same number of euploid and aneuploid blastocysts equally diagnosed between follicular and luteal phase stimulation [39,40]. Regarding the later, it is of interest that they appear to present with the same types of chromosome abnormalities [39]. Conclusively, regarding the DuoStim protocol, various studies evaluated and reported a higher total number of collected oocytes, fertilized oocytes, embryos obtained and, last but not least, euploid embryos [46,49]. Paradoxically, a recent retrospective case-control study, in which double ovarian stimulation was performed in older women, reported more MII and fertilized oocytes, along with the prevalence of better-quality embryos in luteal phase stimulation in comparison to follicular phase stimulation [40]. Nevertheless, this study failed to combine reported results and couple them with a statistical analysis, even though the findings were in accordance with two pilot studies, that reported a higher number of mature oocytes, oocytes collected and number of cleaved embryos [45,50]. This could reduce the cycle cancellation rate in patients with poor ovarian response or of advanced maternal age.

Although the success of the ”DUO stim” protocols has been proven, according to literature [40], there is still a lack of evaluation about the possible alterations of the long-term hormonal profile of these women. It is well documented that the number of days the double-stimulation COS protocols are employed, and the total dosage of gonadotrophins is significantly higher [51]. Higher doses of gonadotropins may negatively affect oocyte quality being associated with recruitment of poorer quality follicles that may otherwise not have been selected in a natural cycle [52,53]. According to a recent meta-analysis, controlled ovarian stimulation has been associated with higher possibilities for preterm birth and lower birth weight [54]. Moreover, these alterations may increase the cost of the IVF treatment as well as exert adverse effects on the patients’ psychology. It has been established that milder IVF treatment as well as less hormone administration reduce the psychological burden of these patients [55,56]. These findings constitute the drivers behind this study and the novelty represented hereby focusing on evaluating the phenomenon of SFW on natural cycles for POR patients excluding the factor of stimulation and all it entails from the equation and comparing the two approaches.

Following oocyte retrieval at the follicular phase and due to the high FSH levels, the patient presents with a second follicular wave [57]. A second follicular wave is indicated considering the high E2 levels recorded and evident through ultrasound evaluation [3]. However, due to high levels of progesterone—following the original ovulation and entering the luteal phase [58]—the SFW fails to lead to ovulation of the luteal phase Graafian follicle. It is evident that progesterone produced by the corpus luteum in luteal phase may contribute to the pituitary suppression through negative feedback, with the consequence of an anovulatory follicular wave [45]. The pituitary suppression combined with the increased E2 levels is possible to lead to cyst formation [59]. Hence, as the hormonal levels do not allow the “relief” attributed to natural ovulation during the luteal phase, it is anticipated that the Graafian follicle will become a cystic follicle at the coexistence of both high E_2_ and progesterone. This unfavorable hormonal state is destined to overthrow efforts and jeopardize a consecutive cycle. A possible management is to wait until the cyst recedes spontaneously. Nevertheless, we should consider the time-demanding nature of this approach [60], as well as the psychological burden experienced by the patients. In light of the above, aspiration of the luteal follicle serves a dual purpose of providing the patient with an extra oocyte, along with assisting with the resumption of the second follicular wave and enabling a better prognosis for the consecutive cycle.

The recruitment of the above phenomenon in IVF practice may provide an improved prognosis for poor responders and patients requiring emergency fertility preservation [49]. The rationale behind the employment of LuPOR is that this practice enables accumulation of more oocytes and viable embryos faster [7]. Optimal management of time and efficient treatment is crucial for women with AMA, POR or cancer patients due to the gonadotoxic effects of the stimulation. These time sensitive patients of advanced age opt for an infertility treatment as their last resort prior to considering egg donation programs [40,46]. The race of collecting as many oocytes as possible in a restricted time frame may be viewed as the holy grail in POR management. It is certain that manipulation of the SFW in clinical practice coupled by LuPOR may majorly assist in addressing both issues: ascertaining treatment in a time competent manner while concurrently maximizing oocyte yield.

At the present study, the aim was to evaluate the oocyte yield, maturity and dynamic of LuPOR oocytes in comparison with the FoPOR oocytes in natural cycles and contribute towards delineating the true place of LuPOR, while evaluating its role in poor responder’s management. This is the first time that natural LuPOR following a natural FoPOR is documented, and, to our knowledge, the first report on the observance of an SFW in a natural menstrual cycle excluding stimulation in poor responders. The only other report on natural cycle LuPOR refers to cancer patients and urgent management of fertility preservation. [41,61].

The comparison between the oocytes retrieved respectively from the two phases within the same menstrual cycle (Table 2) provided no statistical difference in neither the number, the maturity of the oocytes retrieved nor the respective fertilization rate. This report uniquely brings to literature SFW data from natural cycles, which cements the place of LuPOR practice, while in line with previous studies. It seems that the identity of SFW oocytes compares well to follicular phase derived oocytes, a fact which encourages LuPOR practice to be established with safety. Questions remain regarding to whether the similarities reported by these results could extent to account for similarities in the physiology of the oocyte reflecting its true dynamic. Further studies in SFW will undoubtedly provide answers on these concerns.

Combining OR in both phases presented with a statistically significant higher number of oocytes, MII oocytes and 2PN zygotes in comparison to natural FoPORs only (Table 3). The number of lysed oocytes was also higher in LuPOR cycles; however, it did not hinder the increase in 2PN zygotes. This comparison aims to offer insight with respect to the overall management and contribute towards the decision on whether cycles encompassing both follicular phase oocyte retrieval followed by LuPOR are preferable to the standard practice of only FoPOR. The current analysis appears to strengthen the consensus on LuPOR.

Our results are in accordance with current literature on stimulated cycles [62]. Numerous studies have already confirmed that there is no difference between follicular and luteal phase retrieval, regarding the number of mature oocytes collected, the oocyte quality, the fertilization and cleavage rates, along with embryo quality [7,40,48]. LuPOR during a natural cycle has been successfully employed for the urgent management of fertility preservation in cancer patients [41,61]. The analysis on studies focusing on women diagnosed with cancer, who underwent in vitro maturation (IVM) treatment for urgent fertility preservation did not reveal any statistically significant difference in the number of oocytes retrieved and/or cryopreserved, as well as their fertilization rates and number of embryos obtained from the follicular versus the luteal phase [41,61,63]. It may be worth highlighting that, although IVM has been demonstrated as a safe procedure, at least based on preliminary results [59], it is still not considered to be an established assisted reproduction technique, especially for poor responders. The main reason behind this is the lower pregnancy rates in comparison with classic IVF for women over 40 [64], constituting the majority of poor responders. One of the novel findings of this study evaluating LuPOR in POR patients undergoing natural cycles is that this approach may be employed in natural cycles without the need of IVM since there was no statistically significant difference in the number of MII oocytes retrieved in comparison to oocyte retrievals between the two phases.

One major factor that could have a negative influence on the couples’ psychology is the cancellation of cycles in IVF, combined with no oocyte retrieval [65]. In particular, these patients have presented with both physical and psychological burden, stress, anxiety and even depression [66]. In cases of consecutive failures, it is anticipated that extra time will be required in order to overcome this state of mind prior to proceeding with further treatment [65]. Consequently, the time frame required for concluding multiple efforts towards a successful IVF treatment is lengthened. It is imperative that we acknowledge the time sensitive nature of these patients, especially regarding POR women [49]. Adoption of LuPOR as a strategy may allow for time-saving, which in turn exerts a beneficial impact on these patients’ psychology contributing to an overall more efficient treatment.

On the other hand, one must consider the possible complications related to LuPOR practice. Prevalence of the cycles corresponding to empty follicles during LuPOR fails to be reported in current literature. This should be carefully assessed by future studies clinical complications following transvaginal oocyte retrieval ranging from minor vaginal to intraperitoneal bleeding have been extensively described in current literature [66]; however, none of them was identified in our study. What presents as challenging in clinical practice concerning luteal phase oocyte retrieval is the identification and management of the highly vascularized corpus luteum formed in the follicular phase of the same cycle. Irrespective of the fact that studies concur that these complications are rare and most are conservatively managed [66], nonetheless, they should be accounted for especially in the case of LuPOR where OR is anticipated to be performed twice during a menstrual cycle. Further to that, LuPOR patients depending on standard operating protocols may be subjected to anesthesia more than once in a month, an implication that merits investigation on safety and should be included in the equation weighing the benefits versus the disadvantages of the newly implement strategy of LuPOR. However, it should be noted that, regarding this study, the majority of the patients underwent LuPOR without anesthesia. Additionally, this new approach will increase the Assisted Conception Unit’s monthly workload along with the IVF laboratory routine workload on techniques such as vitrification leading to extra storage required for cryopreservation.

In order for complications to be properly assessed, large Randomized Controlled Trials (RCTs) are required and, most importantly, the scientific community practice should allow for adequate time to properly assess advantages and disadvantages, especially in view of the fact that we are navigating through a novel practice. The benefits of LuPOR cycles are unquestionable. The anticipated question is “should this be incorporated as a horizontal application and are we looking towards an era of ‘LuPOR for all’?”. Our data show that the patients that benefit mostly from LuPOR those who really need to invest efficiently their remaining fertile time, portraying women of extreme poor ovarian reserve. According to literature, women presenting with a normal response to COS may also benefit from LuPOR as more oocytes may be obtained on a similar time-frame in comparison to conventional stimulation and oocyte retrieval protocols [67]. Our results indicate that more embryos can be obtained in a certain amount of time when performing LuPOR along with follicular phase OR. This may be crucial for a specific group of patients, classified as time-sensitive. However, there is a clear need for established profile criteria for these infertility patients prior to horizontal application of LuPOR practice delineating which patients truly benefit from it. Future studies should focus on enhancing practice and avoiding inefficient application for these special patients as time and timing is of the essence. Further studies focusing on the time required for a POR patient to achieve a pregnancy employing LuPOR cycles in comparison to non LuPOR cycles may strengthen the characterization of this new practice towards a standard operation procedure (SOP) practice applied horizontally for a specific group of patients.

## 6. Conclusions

The novelty of the present study is reflected through the employment of LuPOR in natural cycles, excluding cancer patients, without the need for IVM. Our results are in accordance with the current literature on stimulation cycles. The emergence of LuPOR may present an important approach in the management of POR patients. The most important parameter for POR patients is time, and LuPOR may assist in yielding a higher number of oocytes during a menstrual cycle. Interestingly, the clinical practice of LuPOR may assist the patient additionally regarding the prognosis of the next cycle, as the oocyte retrieval prevents the development of a cystic follicle due to the high progesterone oestradiol levels already present. Nonetheless, possible complications related to the practice of two oocyte retrievals within one cycle merits further investigation. LuPOR may serve as a novel strategy for the management of poor responders. In order to identify and assess possible benefits and drawbacks related to LuPOR and dismiss or cement, its true place in clinical practice large scale RCTs are imperative.

## Figures and Tables

**Table 1:** Presentation of patients’ age and hormonal levels. Mean values and standard deviations are provided.

	Mean ± SD
Age	44.08 ± 2.24
AMH (ng/mL)	0.88 ± 0.98
FSH (mIU/mL)	17.15 ± 4.86
LH (mIU/mL)	7.11 ± 4.77
E_2_ (pg/mL)	64.09 ± 41.54
Progesterone (ng/mL)	2.38 ± 1.83

AMH: Anti-Mullerian Hormone; FSH: Follicle Stimulating Hormone; LH: Luteneizing Hormone; E_2_: Estradiol; SD: Standard Deviation.

**Table 2:** Comparison between natural Follicular Phase Oocyte Retrievals (FoPORs) and natural Luteal Phase Oocyte Retrievals (LuPORs) during the same menstrual cycle. Mean values ± standard deviation are presented along with median and range for each parameter studied.

		FoPORs				LuPORs			*p*
	Mean ± SD	Median	Range		Mean ± SD	Median	Range		
			Min	Max			Min	Max	
Oocytes Received ^a^	1.22 ± 0.48	**1**	**1**	3	1.29 ± 0.55	**1**	**1**	4	0.30
MII ^a^	0.91 ± 0.45	**1**	**0**	2	1.01 ± 0.48	**1**	**0**	3	0.08
MI ^b^	0.11 ± 0.31	**0**	**0**	1	0.10 ± 0.33	**0**	**0**	1	0.7
GV ^b^	0.11 ± 0.34	**0**	**0**	1	0.12 ± 0.34	**0**	**0**	1	0.84
Abnormal ^b^	0.09 ± 0.28	**0**	**0**	1	0.07 ± 0.26	**0**	**0**	1	0.66
2PN ^a^	0.64 ± 0.56	**1**	**0**	2	0.67 ± 0.54	**1**	**0**	2	0.61
Unfertilized ^b^	0.11 ± 0.31	**0**	**0**	1	0.10 ± 0.34	**0**	**0**	1	0.57
3PN ^b^	0.03 ± 0.19	**0**	**0**	1	0.06 ± 0.23	**0**	**0**	1	0.4
Lysed ^b^	0.07 ± 0.25	**0**	**0**	1	0.08 ± 0.27	**0**	**0**	1	0.64

Values correspond to per retrieval. ^a^: Data followed normal distribution thus the t-test was employed; ^b^: Data did not follow normal distribution thus the Mann-Whitney test was employed; MII: mature oocytes; MI: immature oocytes with no polar body extrusion; GV: immature oocytes with a germinal vesicle; the normally fertilized oocytes bearing two pronuclei (2PN); the number of abnormally fertilized oocytes (3PN).

**Table 3:** Comparison between cycles employing only a natural FoPOR, versus cycles employing both natural FoPOR and subsequent natural LuPOR. Mean value ± standard deviation are presented, along with median and range range for each parameter studied.

		Natural FoPORs Only				Natural FoPORs + LuPORs			*p*
	Mean ± SD	Median	Range		Mean ± SD	Median	Range		
			Min	Max			Min	Max	
Oocytes Received ^a^	1.25 ± 0.53	**1**	**1**	4	2.50 ± 0.78	**3**	**2**	6	<0.001 *
MII ^a^	0.95 ± 0.59	**1**	**0**	3	1.93 ± 0.69	**2**	**0**	5	<0.001 *
MI ^b^	0.11 ± 0.31	**0**	**0**	2	0.21 ± 0.49	**0**	**0**	2	0.08
GV ^b^	0.13 ± 0.33	**0**	**0**	1	0.23 ± 0.50	**0**	**0**	2	0.09
Abnormal ^b^	0.08 ± 0.27	**0**	**0**	1	0.16 ± 0.41	**0**	**0**	2	0.08
2PN ^a^	0.61 ± 0.60	**1**	**0**	2	1.31 ± 0.87	**2**	**0**	4	<0.001 *
Unfertilized ^b^	0.15 ± 0.36	**0**	**0**	1	0.21 ± 0.48	**0**	**0**	2	0.45
3PN ^b^	0.05 ± 0.22	**0**	**0**	1	0.10 ± 0.29	**0**	**0**	1	0.33
Lysed ^b^	0.07 ± 0.26	**0**	**0**	1	0.14 ± 0.35	**0**	**0**	1	0.05 *

* Statistically significant difference. ^a^: Data followed normal distribution thus the t-test was employed; ^b^: Data did not follow normal distribution thus the Mann-Whitney test was employed.
